# Tryptophan Feeding of the IDO1-AhR Axis in Host–Microbial Symbiosis

**DOI:** 10.3389/fimmu.2014.00640

**Published:** 2014-12-15

**Authors:** Teresa Zelante, Rossana Giulietta Iannitti, Francesca Fallarino, Marco Gargaro, Antonella De Luca, Silvia Moretti, Andrea Bartoli, Luigina Romani

**Affiliations:** ^1^Department of Experimental Medicine, University of Perugia, Perugia, Italy

**Keywords:** indoleamine-2,3-dioxygenase, aryl hydrocarbon receptor, resistance, tolerance, microbial symbiosis

The large variety of microbial species in the human microbiome plays an important role in human health by affecting tissue differentiation, modulation of the immune system, as well as the general response against infectious pathogens. The aryl hydrocarbon receptor (AhR) contributes to immune homeostasis as having an antimicrobial role on the one hand – owing to AhR-dependent IL-22 transcription – and, on the other, an anti-inflammatory role in that it mediates the differentiation of regulatory T cells (Tregs). Here, we have examined the multifaceted physiological role of AhR as resulting from the vast array of recently described AhR ligands and of the multiplicity of AhR-expressing cells in host-microbial symbiosis in mammals.

## The Promiscuous Nature of AhR Agonists

Aryl hydrocarbon receptor is a ligand-dependent transcription factor activated by a variety of synthetic and natural molecules. In particular, ligands of AhR include hydrocarbons, heterocyclic amines, and indole-derived compounds. Dioxin (2,3,7,8-tetrachlorodibenzo-*p*-dioxin; TCDD) represents the prototypical environmental and most potent AhR ligand known (1). In addition, a variety of herbal extracts – such as ginseng, licorice, and gingko biloba – stimulate AhR DNA binding and the downstream transcription of numerous genes, thus indicating that AhR might have evolved to respond to mainly dietary products to which animals and humans are chronically exposed (2). Interestingly, ginseng, saponins (gingenosides) have been defined as potent AhR agonists or antagonists (3, 4). Despite the ability of environmental chemicals or other products in diet to bind and subsequently activate AhR, former studies have also shown that natural endogenous ligands may bind and mediate AhR-dependent downstream effects as well (5). Thus the evolution of the AhR – some 450 million years ago – underlies the concept that the original AhR ligands emerged prior to the anthropogenic introduction of polyaromatic hydrocarbons (2). In addition, AhR has an exceptionally promiscuous ligand-binding pocket, which explains the extreme variety of molecules binding AhR with agonist or antagonistic activity (6). Indeed, it has been shown that both bilirubin and biliverdin represent good examples of endogenous ligands in liver with agonist activity for AhR (7, 8). The induction of AhR in the liver by those ligands induces upregulation of *Ugt1a1* to prevent jaundice in neonates and to regulate antioxidant AhR effects in the adult liver (9). The intricacies of AhR activation also relate to the mode of application of a ligand and not only to its nature. Thus systemic administration of 6-formylindolo [3,2-*b*]carbazole (FICZ) reduced clinical signs in a murine model of encephalomyelitis (EAE), while local injection of FICZ, incorporated into the antigen emulsion for induction of EAE, seemed to more directly target and promote Th17 cells, thereby exacerbating pathology in EAE (10).

**Figure 1:**
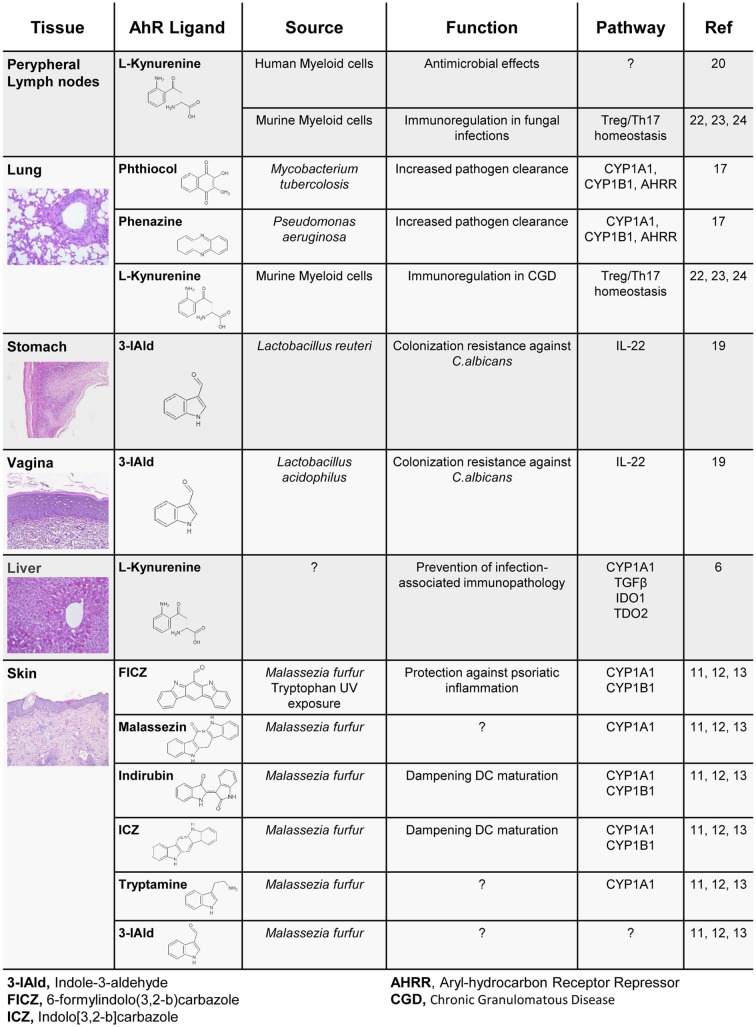
**Tryptophan-derived AhR activating molecules with antimicrobial activity**.

Recently, many studies have shown that the microbiome represents a consistent source of AhR endogenous ligands with disparate effects on immune homeostasis (Figure 1). Thus, moving to the context of the microbiota, the nature of both ligands and target cells vary consistently according to the microbe niche, providing a more complex scenario of AhR’s impact on immune homeostasis. In the human skin, commensals such as *Malassezia* yeasts secrete AhR agonists, such as indirubin, FICZ, indolo[3,2-*b*]carbazole (ICZ), malassezin, pityriacitrin, and tryptamine, which are all potent AhR ligands (11). When skin extracts are isolated from patients with ongoing skin infection, AhR is potently activated, and an increased concentration of AhR ligands in the skin has been linked to the development of *Malassezia*-associated skin diseases. Interestingly, some of the isolated molecules are able to convert to other AhR ligands such as ICZ, which is released under conversion of malassezin (12). Therefore, *Malassezia*-derived AhR ligands may have a significant impact on skin homeostatic immune mechanisms and disease development. Indeed, indirubin and ICZ significantly augmented AhR-mediated *Cyp1a1* and *Cyp1b1* gene expression in dendritic cells, while reducing Toll-like receptor (TLR)-induced dendritic cell maturation and T-lymphocyte proliferation (13). In line with this finding, FICZ has been used to dampen the inflammatory response in both mouse and human skin (14). Through the activation of AhR in non-hematopoietic skin cells, administration of FICZ ameliorated the inflammatory profile of psoriasiform human and murine skin specimens. Of interest, a key aspect of tryptophan-derived metabolites is related to their molecular dynamics of interconversion. For example, tryptamine serves as a proligand for AhR, and its activation depends mainly on monoamine oxidases (15), which eventually convert tryptamine to other AhR ligands, such as the indole-3-aldehyde (3-IAld) and eventually by spontaneous dimerization to FICZ (15). Importantly, intestinal microbiota will also convert tryptophan to tryptamine by decarboxylation. In doing so, and by modulating the colonic ion secretion, tryptamine affects the transit of food particles and bacterial cells through the gut lumen (16). More recently, *Pseudomonas aeruginosa* as well as *Mycobacterium tuberculosis* showed an ability to activate AhR in the lung through the release of pigmented virulence factors, such as phenazines and phthiocol, respectively, pointing to AhR as a sensor of a new class of pathogen-associated molecular patterns. Upon AhR binding, an AhR-controlled metabolic circuit was activated and the virulence factors degraded with consequent pathogen clearance (17).

We found that highly adaptive lactobacilli in the gut, in particular *Lactobacillus reuteri*, by switching from sugar to tryptophan as an energy source, were expanded and produced an AhR ligand, 3-IAld, active in innate lymphoid cells (ILCs) where it would contribute to mucosal resistance against the opportunistic pathogen *Candida albicans*. IL-22 is the main downstream product of AhR activation upon 3-IAld stimulation in ILCs, regulating the release of antimicrobial peptides in the gut epithelia. Of notice, IL-22^+^ ILCs are also able to limit segmented filamentous bacteria colonization in the gut (18). A similar effect was found in the murine vaginal tissue, where *Lactobacillus acidophilus* will degrade tryptophan to 3-IAld and protect mice from *C. albicans* vaginitis (19). Pivotally, these antimicrobial effects were more evident under conditions of higher tryptophan availability in mucosal tissues, as it occurs in mice fed with a tryptophan-enriched diet or in mice bearing deficiency of a tryptophan catabolic enzyme.

In addition to microbial derived ligands, mammalian cells in the liver, as well as in peripheral lymph nodes, activate enzymes such as tryptophan-2,3-dioxygenase (TDO2) and indoleamine-2,3-dioxygenase 1 (IDO1), able to generate tryptophan derivatives such as kynurenines, which also notably act as ligands for AhR (6). Kynurenines have long been known for their ability to exert specific antimicrobial activities (20). Thus, the recent findings provide mechanistic insight into the interplay between IDO1-dependent metabolism and AhR activation in colonization resistance and tolerance induction at the host/microbe interface (6) (Figure 1).

## The IDO1-AhR-Treg Axis in Mammals: The Co-Evolution of a Tolerogenic Defense Strategy

Humans have evolved with microbes, and crucial factors for survival include prompt recognition of invading pathogens, acquisition of controlled immune response, fine-tuned pathogen eradication and return to homeostasis. Co-evolution with hosts had a particularly strong impact on the immune system, which needed to develop an ability to discriminate between resident microbes – maintaining a homeostatic balance – and invasive pathogens, which it must respond to. This complexity could be achieved by integrating two major immune defense mechanisms: infection resistance and disease tolerance (21). Induction of immune tolerance and the maintenance of homeostatic balance provide a series of benefits, including avoidance of tissue injury and para-inflammatory side effects, such as chronic infection and inflammation, which are major epigenetic and environmental factors that contribute to metabolic diseases and autoimmunity, and, in specific settings, to cancer. Conversely, the induction of immune resistance reflects opposite intents, such as the avoidance of infection and control of microbial burden (22, 23).

This paradigm has been epitomized in fungal commensalisms where immune protection must oppose fungal infectivity and ensure survival, while limiting collateral damage and restoring a homeostatic environment (also referred to as “protective tolerance”) (22, 24). Primordial resistance against fungi is mainly mediated by naturally occurring IL-22/IL-17A-producing cells, highly prevalent at mucosal sites, and activated by AhR (19, 25). The tryptophan metabolic pathway appeared to play a key and decisive role in fostering tolerance by means of IDO1 activation, tryptophan starvation, the production of immunomodulatory kynurenines, and the activation of Tregs that are strictly required for the generation of protective tolerance to fungi (26, 27). As AhR activation leads to the activation of IDO1 (25), the regulatory loop involving AhR and IDO1 may have driven the co-evolution of commensal fungi with the mammalian immune system and the microbiota, to the benefit of host survival and fungal commensalism.

More recently, the cross-regulatory circuit between IDO1 and AhR has been elegantly shown to mediate disease tolerance (6). Among IDO1 secondary metabolites, l-kynurenine has been identified as an AhR ligand (6). In turn, the AhR-associated Src activity was responsible for IDO1 phosphorylation, TGF-β production, and Treg cell expansion, thus allowing for endotoxin tolerance to occur. Importantly, the activation of the IDO-AhR-Treg axis prevented *Salmonella typhimurium* infection and significantly reduced clinical signs of *Streptococcus* arthritis (6).

More interestingly, the interaction between IDO1 and AhR may have important roles in the context of autoimmune diseases. Autoimmune diseases are indeed multifactorial, depending on intrinsic or environmental components, including diet, infections, and microbial exposure. The role of AhR in autoimmunity is even more interesting because of evidence on protection to EAE by TCDD, which exerts anti-inflammatory effects through induction of Tregs (1). In this context, it is interesting to note that FICZ also protects against EAE when systemically administered (10). Therefore, future studies are needed to elucidate the possible role of AhR ligands of microbial origin in protecting from autoimmune diseases.

In conclusion, the development of a highly specialized symbiosis requires iterative sets of mutual adaptation between and among symbionts and their hosts. This demands moving beyond surveys of microbial diversity to identify host/microbial metabolites that directly target the IDO1-AhR axis for the promotion of infection control and immune homeostasis.

## Conflict of Interest Statement

The authors declare that the research was conducted in the absence of any commercial or financial relationships that could be construed as a potential conflict of interest.
